# Do People Agree on What Makes One Feel Loved? A Cognitive Psychometric Approach to the Consensus on Felt Love

**DOI:** 10.1371/journal.pone.0152803

**Published:** 2016-04-01

**Authors:** Zita Oravecz, Chelsea Muth, Joachim Vandekerckhove

**Affiliations:** 1 Human Development and Family Studies, The Pennsylvania State University, State College, PA, United States of America; 2 Cognitive Sciences, University of California Irvine, Irvine, CA, United States of America; Hangzhou Normal University, CHINA

## Abstract

This pragmatic study examines love as a mode of communication. Our focus is on the receiver side: what makes an individual feel loved and how *felt love* is defined through daily interactions. Our aim is to explore everyday life scenarios in which people might experience love, and to consider people’s converging and diverging judgments about which scenarios indicate felt love. We apply a cognitive psychometric approach to quantify a receiver’s ability to detect, understand, and know that they are loved. Through crowd-sourcing, we surveyed lay participants about whether various scenarios were indicators of felt love. We thus quantify these responses to make inference about consensus judgments of felt love, measure individual levels of agreement with consensus, and assess individual response styles. More specifically, we (1) derive consensus judgments on felt love; (2) describe its characteristics in qualitative and quantitative terms, (3) explore individual differences in both (a) participant agreement with consensus, and (b) participant judgment when uncertain about shared knowledge, and (4) test whether individual differences can be meaningfully linked to explanatory variables. Results indicate that people converge towards a shared cognitive model of felt love. Conversely, respondents showed heterogeneity in knowledge of consensus, and in dealing with uncertainty. We found that, when facing uncertainty, female respondents and people in relationships more frequently judge scenarios as indicators of felt love. Moreover, respondents from smaller households tend to know more about consensus judgments of felt love, while respondents from larger households are more willing to guess when unsure of consensus.

## Introduction

By adulthood, people develop internal models of social context that consist of sets of cognitive schemata. Such schemata are generalized expectations and preferences regarding relationships that guide interpretation of interpersonal experiences [1]. The research described below uses a novel methodological framework to disentangle the multiple pathways that people expect will elicit loving feelings in others. We provide a complete account of the methodology, including online supplements with computer scripts and the collected data, so that interested researchers can easily implement the proposed methodology for their own research questions or further explore our data.

Empirical work has so far predominantly focused on studying love as a prototypical concept [2, 3], investigating its taxonomies [4] or tracing its biological roots [5, 6]. Research questions most often center on romantic love, [7–9] and most measurement instruments are also typically developed for romantic or passionate love [10, 11].

Our line of inquiry focuses on interpersonal relationships in the context of well-being: we investigate the feeling of being loved from the perspective that felt love is a component of everyday well-being. We introduce the concept of felt love in the context of our overarching goal to measure multidimensional elements of well-being—including positive relationships, positive emotions, engagement, and accomplishment—in everyday life.

Felt love may be defined as the degree to which an individual is receptive to loving signals in interpersonal interactions. To measure a person’s level of felt love, we must be able to answer the question: what makes people feel love? Our pragmatic approach to measuring felt love may be thought of in terms of love communication, i.e. the sending and receiving of love signals. In the current project, we approach felt love as part of a communication process in which a sender projects their affection, through some possibly noisy medium, and a receiver interprets these signals. The interpretation, in turn, generates the internal feeling of being loved in the receiver. To succeed, such communication requires consensus on which actions express love—just as verbal communication requires consensus on words and their meanings. As a consequence, knowledge of *shared* beliefs—i.e., cultural ideas and definitions of loving actions—play a crucial role in a person’s ability to feel love. The felt love survey implemented in this study (see Study settings, below) serves as a tool for capturing how accurately a person receives culturally consensual love signals. It is important to clarify that this tool simultaneously measures an individual’s level of felt love and the underlying population level (cultural consensus) of felt love towards the same signal.

According to adult attachment theory, [12–14] worries about the quality of our interpersonal relationships evoke anxiety that shapes our adult attachment style. In the study below, the cognitive aspects of this feeling are brought to focus by exploring overlaps in people’s judgments of when one is expected to perceive love. While some well-being measures [15] already include questions such as “How much do you feel loved,” the specific elements and examples that conjure felt love have not been systematically studied. With our proposed methodology we aggregate information from lay judgments to derive a cultural consensus on “felt love.”

Cultural consensus theory [16] (CCT) is a cognitive psychometric [17, 18] approach to information aggregation. CCT models are able to derive shared agreement or “consensus truth” from sets of items centered on a knowledge domain, while simultaneously accounting for and measuring differences in knowledge levels and cognitive response biases of respondents. These models have been extensively applied in anthropology (for example to study medical knowledge and beliefs [19]), have been effective for extracting information from eye-witness testimonies [20], have been used for inferring judgment of personality traits in social networks [21, 22], and were recently proposed for evaluating interpersonal agreements on theoretical concepts [23] in psychology.

The goal of the present paper is to study the concept of felt love as it manifests in everyday life interactions. Our central intuition is that we can investigate people’s cognitive models of scenarios that elicit love, first by asking lay participants to generate descriptors of felt love situations, and then by asking a second participant group to evaluate a list of these possible felt love situations, derived from the first subjects and a literature review. In order to better understand felt love, how people differ in knowing about shared agreements of felt love, and how people differ in their responses to questions on the topic, our analysis uses a cognitive psychometric model of consensus in the CCT framework (described above). The main advantage of the proposed approach is that when it comes to inferring shared agreements of felt love from observed and potentially noisy data, interpersonal and inter-item differences can be taken into account to improve accuracy of inference. While individual differences discovered in this process are interesting in their own right, they have potential to be further linked to covariates such as gender or age, and advance our understanding of the mechanisms related to felt love.

The paper is structured as follows: First, the Methods section describes the research methodology with a substantiation of why it is ideal for studying felt love. Then we show a set of items describing situations in which people might feel loved, derived from literature review and lay people’s opinions. These items were then used to test whether individuals agree that people should feel loved in specific situations, and whether there are individual differences in their consensus knowledge level and cognitive response characteristics. These analyses facilitate inference, not only about shared agreement on items, but also about rank ordering of everyday felt love situations based on the difficulty people have in being aware of the cultural consensus for each item. The Result section gives a summary of these findings.

## Methods

The study presented below was approved by the University of California, Irvine, Office of Research, Institutional Review Board, under HS# 2013–9918. Data were collected via Amazon Mechanical Turk (MTurk). Participants indicated consent on the website.

We compiled an item bank with 53 items describing scenarios in which people might feel loved. All these items tap into the collective knowledge pool. Respondents evaluated these questions in terms of True/False/Don’t know. Consensus models were then used to quantify the extent of consensus and influencing factors as related to a shared knowledge domain. The following subsections elaborate on the methodological steps.

### Study settings

The felt love questionnaire contains a collection of 53 items each describing a scenario in which people might feel loved. These items (together with some results) are listed in S1 Table in the Supporting Information. All items started with “Most people feel loved when …”. The second part of the items loosely clustered by topics including: *support in needs and goals* (e.g., “someone celebrates their accomplishments”), *sharing time with others* (e.g., “they spend time with their friends”), *trust and acceptance* (e.g., “when somebody confides in them”), *symbolic/physical expressions* (e.g., “they get gifts”), *other possible sources of love* (e.g., religion, pets, nature, patriotism, gratitude, politeness, etc.), *controlling behavior from others* (e.g., “someone tries to change their behavior to be healthier”), *neutral items* (e.g., “they eat their favorite food”).

We used Amazon Mechanical Turk [24] (MTurk) to collect responses from the general population. MTurk is a crowdsourcing website that allows to reach a diverse group of people. Using MTurk, we recruited 150 participants (the number for which we had budgeted). Participants indicated consent on the website. In addition to the 53 felt love items we also included basic demographic questions, together with two questions eliciting open ended feedback, one about the survey in general and one asking about potential additional items. The sample consisted of 57.33% men, 42% women (1 person preferred not to specify gender), with mean age 33.29 (SD = 10.28). The ethnic composition of the sample was the following: 70.67% white, 8% black, 13.33% Asian or Pacific Islander, 5.33% Hispanic or Latino (with the remaining 2.6% falling in other categories or preferred not to answer). One respondent did not have a high school degree, 32% of the respondents had high school degrees, 19% had college degrees and the rest of the respondents (46.66%) had bachelor degrees or higher (with 2 people opting for no answer on this question). To ensure quality responses, we collected responses only from workers with “master badges” (a quality check on each worker’s response created by Amazon). All data and scripts used for the analysis can be downloaded from https://github.com/zoravecz/FeltLovePLOSONE.git. The respondents first responded to the demographic questions, then evaluated the 53 items in terms of True/False/Don’t know, then were asked to give feedback.

### The Extended Condorcet Model: a cognitive psychometric model to derive consensus

In this study it is reasonable to assume that some people might be more aware of the meaning of interpersonal actions in everyday life scenarios. The proposed consensus model allows for individual differences in the degree to which a respondent is in agreement with others. Moreover, when responding in the ternary terms of True/False/Don’t know, some respondents are more likely to guess when uncertain, whereas others are more likely to mark “Don’t know” [25]. Guessing tendencies have similarly been shown to differ between individuals [26].

**Fig 1:**
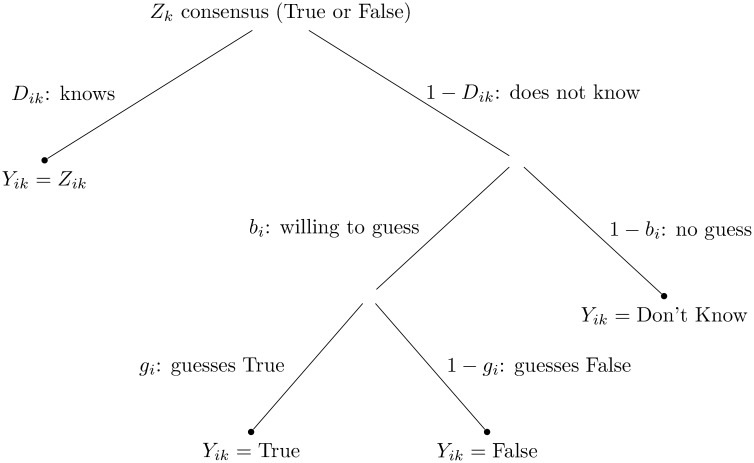
Processing tree depiction of the Extended Condorcet Model.

In order to derive the shared agreement (i.e., consensus truth) on the 53 felt love items, all these individual differences have to be factored in and the dependence structure in of the data (items centering around the same concepts, respondents sharing the same cultural background) should be accounted for. The consensus model we propose captures these factors through a multinomial processing tree model shown in Fig 1. This model is dubbed the Extended Condorcet Model [27] (ECM).

*N* = 150 respondents answered *M* = 53 items. The answer from a single respondent *i* (*i* = 1, …, *N*), for an item *k* (*k* = 1, …, *M*) is denoted *Y*_*ik*_. On the data level there are three answer categories, ‘True’, ‘False’ and ‘Don’t know’. Fig 1 summarizes the decision tree of a single respondent *i* for item *k* based on the proposed cognitive model for the ECM. The consensus answer (cultural truth) is denoted *Z*_*k*_ for item *k*. The probability that respondent *i* knows the consensus answer for item *k* is *D*_*ik*_. The probability that respondent *i* will venture a guess is *b*_*i*_. If the respondent is not willing to guess, it is assumed that they mark the ‘Don’t know’ option, with probability 1−*b*_*i*_, in which case they would respond ‘True’ with probability *g*_*i*_.

The probability for each answer category can be derived by following the branches of the corresponding answers in Fig 1. Then the likelihood is formulated as a categorical distribution with a probability vector that collects the three probabilities for the three response options (see p. 195, [27]). Furthermore, the probability of knowing the consensus truth (*D*_*ik*_) for a certain person *i* for a given item *k* is decomposed into (1) the person’s latent ability or knowledge level, *θ*_*i*_, as relating to the domain (in our case knowing in which situations most people would feel loved) and (2) the item’s difficulty, *δ*_*k*_, such as

$$\begin{array}{c} D_{ik}={\text{logit}}^{-1}\left(\theta_{i}-\delta_{k}\right), \end{array}$$ (1)

where logit^−1^ stands for the inverse logit or logistic function. The item’s difficulty level *δ*_*k*_ represents how hard it is to know the consensus answer for that given item. The logistic function maps values on the real line to the unit (probability) scale. For example, when the respondent’s dominance over the item (the difference between *θ*_*i*_ and *δ*_*k*_) is zero, *D*_*ik*_, the probability of knowing the consensus, equals 0.5. If the respondent *i*’s ability *θ*_*i*_ is greater than the item’s difficulty *δ*_*k*_, then the respondent has a better-than-even probability (*D*_*ik*_ > 0.5) of knowing the consensus for item *k*.

As mentioned above, our interest lies not only in describing and accounting for meaningful individual differences, but also potentially tying them to interesting person-specific aspects, such as gender and age. Therefore explanatory variables (covariates) about the respondents are also added to the model. As seen in the above description, the observed data is modeled via psychologically interesting parameters, and we will be able to tie covariates like gender, relationship status etc. to these separate aspects of cognitive functioning.

### Model fitting in the Bayesian statistical framework

Consensus models have gained considerable momentum since recently being implemented in the Bayesian statistical framework.[28–30] Bayesian methods [31] provide flexible tools for probabilistic modeling, together with principled ways of statistical inference. More specifically, Bayesian modeling offers a straightforward way to quantify uncertainty in information, and Bayesian inference is consistent for any sample size. The complexity of the studied phenomenon can be best answered by models that allow person-specific differences in all the leading cognitive model parameters. Complex hierarchical cognitive models like the consensus model presented above could not be fitted in the classical statistical framework.

We fit the Extended Condorcet Model in the Bayesian statistical framework. Bayesian parameter estimation is carried out by deriving the posterior probability distribution of the parameter. This posterior probability distribution is proportional to the product of some prior distribution defined before seeing the data and the likelihood function, which is a formal mathematical description of the assumed decision process generating the observed data. In both models below so-called non-informative prior distribution are used, which means that we did not assume prior information regarding any of the model parameters. The likelihood is the probabilistic formulation of the ECM described in the previous section.

Some level of modeling complexity is required to be able to adequately address the relationship between individual differences in the cognitive parameters of the ECM and explanatory variables. For example if we want to study whether individual differences in the knowledge on felt love can be tied to characteristics such as gender and age, our dependent variable is a model parameter. This can be formalized by the following equation:

$$\begin{array}{c} \theta_{i}=\beta_{0}+\beta_{1}*{\text{gender}}_{i}+\beta_{2}*{\text{age}}_{i}. \end{array}$$ (2)

As can be seen in Eq 2, it is not the observed data that is made function of explanatory variables (in this example gender and age), as is commonly done in standard regression analysis. In our framework, the dependent variable (left hand-side) is a meaningful person-specific cognitive model parameter. The Bayesian framework allows us to estimate all model parameters in a one-step procedure. That is to say, the posterior distribution of consensus truth, person and item parameters, variance parameters and regression coefficients are derived at once. If we first estimated the person-specific model parameters only, and in a second step regressed some point estimates of these parameter (e.g., the person’s knowledge on felt love) on the explanatory variables, the uncertainty in the model parameter estimates that we get in the first step would be neglected, leading to an overestimation of the standard error of the regression coefficients. Our proposed one-step procedure is useful for avoiding this so-called generated regressor bias [32]. Fitting the consensus model in one-step within the Bayesian statistical framework allows uncertainty in the estimates to be taken into account in a consistent and straightforward way and provides a probabilistic description of the possible values for each parameter in terms of posterior probability distributions. For a general introduction on hierarchical/multilevel models, see Gelman and Hill [33] and on random effects and predictors on latent variables, see De Boeck and Wilson [34]. For another example on the combination of cognitive models and latent variables, see a study on the joint analysis of behavioral and personality data [35].

## Results

We fitted the consensus model to the data on felt love in JAGS (“Just Another Gibbs Sampler” [36]) by running 8 chains, 1000 burn-in, and 1000 iterations. Convergence of the 8 chains was tested in both models by the $\hat{R}$ statistics, and confirmed using the standard criterion that the estimated potential scale reduction is $\hat{R}<1.1$. MATLAB and JAGS scripts to re-run the analysis are also included in the shared public GitHub folder indicated above.

### Consensus estimates on felt love items from the ECM

Table 1 contains raw data scores and model parameter estimates on some selected felt love items. The first column contains the item descriptions, the second column is the average raw score for that item with True responses coded as 1 and False responses as 0. The derived consensus on felt love in terms of posterior median estimates (labeled as False for 0 and 1 for True) for each item are displayed in column 3. In the next column, the posterior standard deviations quantify the uncertainty in these median point estimates. Finally, column 5 shows the item difficulty rank for each scenario. Number 1 indicates the most difficult item to judge the consensus on, which means that only people with very high levels of knowledge about the shared agreement on felt love are likely to get it right.

**Table 1:** Raw data means and model based estimates on selected felt love items. The second columns shows the mean of the answers for the item with ‘True’ coded as 1 and ‘False’ as 0. The posterior distribution on the consensus parameters for each item is summarized in columns 3 and 4, in terms of posterior median estimate, labeled as ‘True’ for 1 and ‘False’ for 0 and posterior standard deviation (abbreviated as ‘psd’). It quantifies standard error around the point estimate. The last column shows the item difficulty rank of the item in ascending order.

		T/F	consensus item diff.		
nr	Most people feel loved when …	mean	label	psd	rank
20	someone cares for them	0.97	True	0.02	53
22	their pets are happy to see them	0.97	True	0.03	52
27	they are made to feel special	0.96	True	0.34	51
36	they are told that they are loved	0.95	True	0.46	49
01	someone supports them without expecting anything in return	0.94	True	0.00	50
23	they attend a religious ceremony	0.51	True	0.02	3
49	they feel close to nature	0.46	False	0.49	1
26	someone wants to know where they are at all times	0.44	False	0.03	4
44	someone is polite to them	0.38	False	0.42	2
14	someone is sexually attracted to them	0.62	True	0.00	6

The first half of the items in Table 1 show scenarios for which people converged toward a consensus easily. This is apparent on the raw data level as well: column 3 shows that almost all respondents endorsed these items. The consensus estimate, labeled ‘True’ or ‘False’ turns out to be ‘True’ for all of these items in column 4. As can be seen, the posterior standard deviation in column 5 is 0, indicating virtually no uncertainty in these posterior point estimate. From the last column we can learn that these five scenarios are the easiest items (there were 53 items in total).

For the second half of Table 1, we selected five items for which the raw data showed the highest amount of split (i.e., the number of True and False responses were really close). As can be seen, sometimes people were almost evenly split on these items. In these cases, consensus modeling is especially useful: by working with the full matrix of trichotomous responses instead of only row marginals of True and False responses, we are able to aggregate information across items while also accounting for individual differences in terms of cognitive parameters. That is, if we were to take the average of observed responses from all participants for a specific item, we would neglect the fact that the rest of their responses in the matrix are on similar items (all items are on felt love, hence they are not independent). Also, such an averaging strategy would neglect that some responses might come from guessing. Moreover, the Don’t know responses would have to be treated as missing data even though it is reasonable to assume that these are missing not at random. The model based consensus answer on an item takes into account all these relations between the data points. Furthermore, it captures possible heterogeneity of the items (for some scenarios it might be more difficult to know the consensus), while it also allows the participants to differ in their response style and knowledge level. As a result, analyzing the data with the consensus model provides more information than simple summary statistics.

The item with the highest split was about people feeling loved when they attend a religious ceremony. The second row of Table 1 shows another item with a relatively high uncertainty based on the raw data. If we compare the posterior standard deviations however, only the “close to nature” item has a relatively high uncertainty (posterior standard deviation is 0.49) in its consensus estimate, which is inferred to be “False”. In contrast, the “attending a religious ceremony” item has very low uncertainty (posterior standard deviation is 0.02), and the consensus is “True” on this item. The difference in terms of uncertainty in these estimates is due to the fact that for the “attending a religious ceremony” item the model was able to borrow enough information from other sources than just the raw answers to this particular item. The most likely contributing factor was the ability level of those who answered “True” to this item. When it comes to the “attending a religious ceremony” scenario, most likely there was no such pattern. This indicates that people do not converge to a general agreement and could be split into two different “cultures” based on this question. However, for most of the 53 items the posterior uncertainty is 0, for items 20 and 22 it is very low; and the only other item with high uncertainty is item 44 (“someone is polite”), which has posterior standard deviation 0.42. Therefore, it might not be so meaningful to conclude that there are two underlying cultures on felt love because people are clearly split about their opinion on nature, and somewhat on politeness. The posterior predictive check (see below) provides further evidence that the one-underlying-culture model is a good fit. The other two items from the second half of the table were estimated to have strong consensus: people do not think that controlling behavior, such as someone wanting to know where they are at all times is a loving signal, while sexual attraction tend to make people feel loved. While exploring the consensus judgment in details on all 53 items falls outside of the scope of the current paper, we invite the reader to browse these interesting substantive results by consulting S1 Table.

### Individual differences in consensus knowledge level and cognitive biases related to felt love

**Fig 2:**
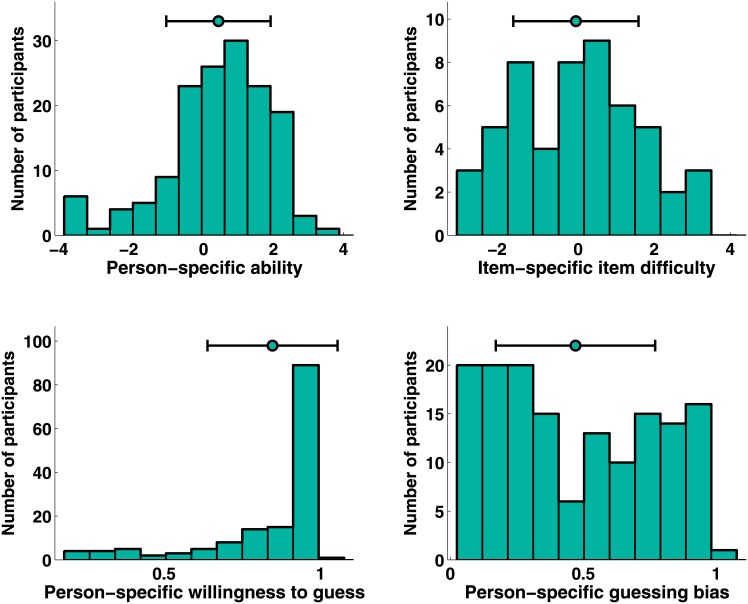
Distribution of person- and item-specific Extended Condorcet Model parameter estimates. Frequency of posterior mean estimates. The middle of the bar indicates their mean; The end points are 1 standard deviation away.

In the current study people showed considerable amount of individual differences in their cognitive characteristics. Fitting the consensus model to the data allows us to summarize individual differences in terms of three parameters: a latent person-specific ability, a person-specific guessing (acquiescence) bias (probability of guessing true) and the willingness to guess. Fig 2 illustrates the heterogeneity in the population by summarizing the frequency person-specific posterior mean parameter estimates in histograms.

In the first row the plot on the left hand side shows the distribution of the 150 person-specific ability estimates. These quantify how well respondents know the consensus on felt love. We can interpret these values in relation to item difficulties. Estimated item difficulty in the current study are displayed in the right slide plot of row 1. As formalized in Eq 1, the probability of being right on a specific item is a function of the person’s ability and the item’s difficulty. The average item difficulty is fixed to 0 and the scale typically ranges from −4 to 4. This is a standard practice to identify the simplest item response theory model, please consult the literature on explanatory item response modeling for more details [34]. Therefore a person whose ability is estimated to be 0 would get an average item right about half of the time. A person, whose ability is around 2 would get an average difficulty item right most of the time, and would have a good chance to know a more difficulty item as well, and so on. In the current study most people had above average level of ability, so they would have a good chance to get the consensus right. This means that we had a good level of ‘signal’ in the data. The middle of the bar above the person-specific ability estimates shows the mean of the plotted values (*μ* = 0.43), and the end points of it correspond to one standard deviation (*σ* = 1.49) above and one below this mean. From the item difficulty plot on the right hand side we can see that the 53 items covered a satisfactory range of easy, medium and difficult items (*μ* = −0.05, *σ* = 1.65).

The second row shows the distribution of the posterior point estimates of the person-specific parameters that are related to cognitive response style. Both parameters are probability parameters, that is their range is between 0 and 1 with higher values being more likely. As can be seen from the left hand side plot, most people were willing to guess when they were unsure, as their estimated willingness to guess probability values are close to 1 (*μ* = 0.85, *σ* = 0.21). This was suggested in the observed data by the low number of “Don’t know” responses as well (around 5% of all responses). However, we can see that there were some individual differences in this characteristic as well, since a handful of people were described by really low willingness to guess tendencies.

With respect to the person-specific guessing bias estimates in the right hand side plot of the second row, values close to 1 correspond to person profiles with a tendency of guessing ‘True’ most of the time, values in the middle represent no systematic guessing tendency and values close to 0 mean guessing ‘False’ most of the time. It appears that respondents show high level of heterogeneity in this sense with the majority of them somewhat more likely to guess ‘False’ when they did not know the consensus answer (*μ* = 0.47, *σ* = 0.30).

### Links between individual differences and explanatory variables

In the Bayesian context all regression coefficients have posterior probability distributions. One way to draw inference about regression coefficients is to compute the probability that it lies away from 0; In other words: is much of the posterior mass isolated to one side of 0 or not? In Table 2 we summarized results on those explanatory variables for which it was at least 95% likely that their estimated value is above or 95% likely that it is below 0. We had four regression coefficients like this (not counting the intercepts). Results on the remaining regression coefficients (18 in total) are given in S2 Table in the Supporting Information. All explanatory variables were standardized for ease of interpretation.

**Table 2:** Summary of the most relevant explanatory variables. The first column shows the ECM parameter name, second column is the explanatory variable name, third column is the posterior mean estimate of the corresponding regression coefficient, fourth column is its posterior standard deviation and the last column shows the probability of the regression coefficient being smaller than 0.

Parameter	Predictor	mean	psd	p(<0)
Consensus knowledge	Household size	−0.27	0.15	0.96
Guessing “True”	Gender (1: male)	−0.46	0.22	0.98
Guessing “True”	In a relationship	0.41	0.22	0.03
Willingness to guess	Household size	0.50	0.28	0.03

As mentioned above, each regression coefficient estimate has a posterior distribution that summarizes ranges of plausible values in terms of probabilities. The third column of Table 2 shows the mean of this distribution, the fourth column shows the standard deviation, and the last column summarizes the probability that the plausible range for this parameter is below 0. In this last column, values close to 1 indicate substantial probability that the likely values for this parameter are negative: for example when consensus knowledge predicted from household size, the probability of the regression coefficient being negative is 0.96. The mean estimate in the same row shows the size of this predictive link (−0.27). Values close to 0 in the last column indicate small probabilities of the coefficient being negative, which in turn means large probabilities of the regression coefficient being positive.

People who live with more family members (*μ* = 1.82, *σ* = 1.66) in the same household seem to know the consensus on felt love less well, however, they seem willing to guess. This seems to suggest that people who are living with others feel that they have a good idea in which situations people experience love and they hardly ever mark “Don’t know”; however, they might have some systematic biases, as they tend to have a lower level of consensus knowledge. Male respondents were less likely to guess “True” when they were not sure about the consensus answer. However, respondents who were in a relationship were more likely perceive scenarios as loving ones when they were unsure.

### Model fit: posterior predictive check of whether respondents converge towards consensus on felt love

In the Bayesian framework absolute model fit can be performed via posterior predictive model checking (PPC). For that we first select a statistical summary that reflects an important feature of the real data. Based on the ECM and the posterior distribution we generate several hundred new data sets. Then we calculate the selected statistical summary statistic on each of these new data sets and on the observed data; then we compare the two. If the summary statistics based on the observed data do not appear to be consistent with the distribution of statistics generated from the replicated data, it is unlikely that the proposed model provides a good description of the observed data.

We used a statistical summary measure developed in the framework of Cultural Consensus Theory [37]. They proposed to calculate eigenvalues from the respondent-by-respondent correlations based on the respondents’ answers. Such a measure is analogous to the indicator of the possible factor solutions in factor analysis: it allows for testing whether the first factor accounts for most of the variation among the correlated variables, and the other factors are simply fitting noise in the data. A one-factor solution is expected to have its first eigenvalue multiples of the second one. In our case, a one-factor solution means that people converge towards a single consensus truth on the items, that is to say that they share an agreement on the signs of felt love.

**Fig 3:**
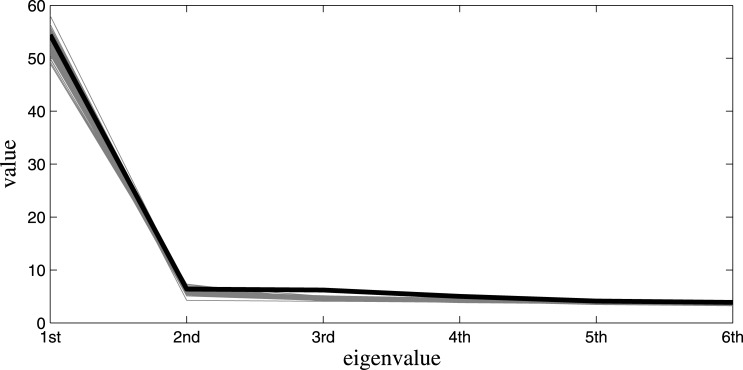
Posterior Predictive Check of One Underlying Consensus Answer Key. Black line: eigenvalues of real data. Thin lines: eigenvalues based on generated data sets.

The black line in Fig 3 shows the eigenvalues calculated from the real data. The area designated by the gray lines represent eigenvalues extracted from 500 replicated data sets based on the model and the posterior distribution of the parameters. The sharp decline between the first and second eigenvalues in every line in Fig 3 suggests that there is one dominant consensus solution in every data set. The rest of the eigenvalues practically follow a straight line, indicating no meaningful difference after this first factor is extracted. We conclude that the model is replicating this essential data property well.

### Discussion

The many true items in this study showed that people see love as something more than can be experienced in romantic settings: a feeling that pervades our everyday life. From looking at the inferred consensus on the full set of items, it turns out that receiving a compliment, appreciation, a gift, or generally an act of kindness (items 34, 10, 15, 46) are all loving signals for most people, and these might arise in any kind of interpersonal interaction from day to day.

While people clearly converged in their beliefs on felt love, the presented study also showed important sources of individual differences. We explored possible ties between the sources of this heterogeneity and person-specific characteristics via regressing psychologically meaningful model parameters on explanatory variables such as age and gender. However, we consider this mainly as methodological demonstration and we acknowledge that the complexity of the subject can be better addressed by a more carefully selected set of explanatory variables, including measures of personality traits.

To the best of our knowledge current scientific literature lacks systematic and methodologically rigorous focus on what it means to feel loved and how people experience it. We showed how state-of-the-art cognitive psychometric techniques implemented in the hierarchical Bayesian framework can help answer complex questions related to felt love. For example, we show how statistical modeling can be used to show that there is agreement among people on what loving signs are (low posterior standard error, posterior predictive check). We see the presented research as a starting point to develop a consistent methodological background that can help study complex phenomenon of experiencing love in a scientifically rigorous way. With respect to potential future directions, we consider it especially interesting to study how feeling loved relates to experienced and evaluated well-being, but other possible future questions can include investigating for example how felt love is related to attachment styles.

## Supporting Information

S1 Table A summary of the raw data and Extended Condorcet Model based estimates. (PDF)

S2 Table Model parameters regressed on a set of predictors. (PDF)

## References

[1] Lopez FG. The assessment of adult attachment security. American Psychological Association; 2003.

[2] Aron A, Fisher HE, Strong G. Romantic love The Cambridge handbook of personal relationships. 2006; p. 595–614.

[3] Fehr B, Sternberg R. A prototype approach to studying love The new psychology of love. 2006; p. 225–246.

[4] Berscheid E. Searching for the meaning of “love” The new psychology of love. 2006; p. 171–183.

[5] Buss DM. The evolution of love The new psychology of love. 2006; p. 65–86.

[6] Fisher H. The drive to love: The neural mechanism for mate selection The new psychology of love. 2006; p. 87–115.

[7] Acevedo BP, Aron A, Fisher HE, Brown LL. Neural correlates of long-term intense romantic love Social cognitive and affective neuroscience. 2011; p. nsq092. 10.1093/scan/nsq092 PMC3277362 21208991

[8] Gonzaga GC, Turner RA, Keltner D, Campos B, Altemus M. Romantic love and sexual desire in close relationships. Emotion. 2006;6(2):163 10.1037/1528-3542.6.2.163 16768550

[9] Hazan C, Shaver P. Romantic love conceptualized as an attachment process. Journal of personality and social psychology. 1987;52(3):511 10.1037/0022-3514.52.3.511 3572722

[10] Rubin Z. Measurement of romantic love. Journal of personality and social psychology. 1970;16(2):265 10.1037/h0029841 5479131

[11] Hatfield E, Bensman L, Rapson RL. A brief history of social scientists’ attempts to measure passionate love. Journal of Social and Personal Relationships. 2012;29(2):143–164. 10.1177/0265407511431055

[12] Fraley RC, Shaver PR. Adult romantic attachment: Theoretical developments, emerging controversies, and unanswered questions. Review of general psychology. 2000;4(2):132 10.1037/1089-2680.4.2.132

[13] Mikulincer M, Shaver PR, Sapir-Lavid Y, Avihou-Kanza N. What’s inside the minds of securely and insecurely attached people? The secure-base script and its associations with attachment-style dimensions. Journal of personality and social psychology. 2009;97(4):615 10.1037/a0015649 19785482

[14] Mikulincer M, Shaver PR.Attachment in adulthood: Structure, dynamics, and change. Guilford Press; 2010.

[15] Kern M, Waters L, Adler A, White M. A multifaceted approach to measuring wellbeing in students: Application of the PERMA framework. Journal of Positive Psychology, available as on-line first. 2014;. 10.1080/17439760.2014.936962 PMC4337659 25745508

[16] Romney AK, Batchelder WH. Cultural Consensus Theory In: Wilson R, Keil F, editors. The MIT encyclopedia of the cognitive sciences. Cambridge, MA: The MIT Press; 1999 p. 208–209.

[17] Batchelder WH. Cognitive psychometrics: Combining two psychological traditions; 2007 CSCA Lecture, Amsterdam, The Netherlands.

[18] van der Maas HL, Molenaar D, Maris G, Kievit RA, Borsboom D. Cognitive psychology meets psychometric theory: on the relation between process models for decision making and latent variable models for individual differences. Psychological review. 2011;118(2):339 10.1037/a0022749 21401290

[19] Weller SC, Baer RD, de Alba Garcia JG, Javier E, Rochad ALS. Explanatory models of diabetes in the U.S. and Mexico: The patient-provider gap and cultural competence. Social Science & Medicine. 2012;75 (6):1088–1096. 10.1016/j.socscimed.2012.05.003 22703883

[20] Waubert de Puiseau B, Aßfalg A, Erdfelder E, Bernstein DM. Extracting the truth from conflicting eyewitness reports: A formal modeling approach. Journal of experimental psychology: applied. 2012;18(4):390 23088437 10.1037/a0029801

[21] Batchelder WH, Kumbasar E, Boyd JP. Consensus analysis of three-way social network data. Journal of Mathematical Sociology. 1997;22:29–58. 10.1080/0022250X.1997.9990193

[22] Agrawal K, Batchelder WH. Cultural Consensus Theory: Aggregating Signed Graphs Under a Balance Constraint In: Shanchieh JY, Greenberg AM, Endsley M, editors. Social Computing, Behavioral-Cultural Modeling, & Prediction (SBP 2012). Heidelberg: Springer; 2012 p. 53–60.

[23] Oravecz Z, Faust K, Batchelder WH. Studying the existence and attributes of consensus on psychological concepts by a cognitive psychometric model. American Journal of Psychology. 2015;128:61–75. 10.5406/amerjpsyc.128.1.0061 26219174

[24] Buhrmester M, Kwang T, Gosling SD. Amazon’s Mechanical Turk a new source of inexpensive, yet high-quality, data? Perspectives on psychological science. 2011;6(1):3–5. 10.1177/1745691610393980 26162106

[25] Mondak JJ. Developing valid knowledge scales. American Journal of Political Science. 2001; p. 224–238. 10.2307/2669369

[26] Plant EA, Devine PG. Internal and external motivation to respond without prejudice. Journal of personality and social psychology. 1998;75(3):811 10.1037/0022-3514.75.3.811

[27] Oravecz Z, Faust K, Batchelder WH. An extended Cultural Consensus Theory model to account for cognitive processes in decision making in social surveys. Sociological Methodology. 2014;44:185–228. 10.1177/0081175014529767

[28] Anders R, Batchelder WH. Cultural consensus theory for multiple consensus truths. Journal of Mathematical Psychology. 2012;56(6):452–469. 10.1016/j.jmp.2013.01.004

[29] Oravecz Z, Anders R, Batchelder WH. Hierarchical Bayesian modeling for test theory without an answer key. Psychometrika. 2015;80:341–367. 10.1007/s11336-013-9379-4 24327065

[30] Oravecz Z, Vandekerckhove J, Batchelder WH. Bayesian cultural consensus theory. Field Methods. 2014; p. 207–222. 10.1177/1525822X13520280

[31] Gelman A, Carlin JB, Stern HS, Rubin DB. Bayesian data analysis. vol. 2 Taylor & Francis; 2014.

[32] Pagan A. Econometric issues in the analysis of regressions with generated regressors International Economic Review. 1984; p.221–247.

[33] Gelman A, Hill J, et al Data analysis using regression and multilevel/hierarchical models. Cambridge University Press; 2007.

[34] De Boeck P, Wilson M. Explanatory Item Response Models: A Generalized Linear and Nonlinear Approach. New York: Springer; 2004.

[35] Vandekerckhove J. A cognitive latent variable model for the simultaneous analysis of behavioral and personality data. Journal of Mathematical Psychology. 2014;60:58–71. 10.1016/j.jmp.2014.06.004

[36] Plummer M, et al JAGS: A program for analysis of Bayesian graphical models using Gibbs sampling In: Proceedings of the 3rd international workshop on distributed statistical computing. vol. 124 Technische Universit at Wien; 2003 p. 125.

[37] Batchelder WH, Anders R. Cultural consensus theory: Comparing different concepts of cultural truth. Journal of Mathematical Psychology. 2012;56(5):316–332. 10.1016/j.jmp.2012.06.002

