# Supplementary material for: Do People Agree on What Makes One Feel Loved? A Cognitive Psychometric Approach to the Consensus on Felt Love
Source: PLoS One. 2016 Apr 1;11(4):e0152803. doi: 10.1371/journal.pone.0152803 (PMC4818109; doi:10.1371/journal.pone.0152803)
Supplement: S1 Table — (PDF) [file pone.0152803.s001.pdf]

Do people agree on what makes one feel loved? A cognitive psychometric approach to the consensus on felt love

Zita Oravecz[1, *], Chelsea Muth[1], and Joachim Vandekerckhove[2]

1 Human Development and Family Studies, The Pennsylvania State University, State College, PA, USA
2 Cognitive Sciences, University of California, Irvine, Irvine, CA, USA

*** zita@psu.edu**

Supporting Information

S1 Table

Table. A summary of the raw data and Extended Condorcet Model based estimates.

S1 Table. Raw data summary and Extended Condorcet Model based estimates on the 53 felt love items.

nr	Most people feel loved when ...	T/F mean	consensus label	consensus psd	item diff.
1	someone supports them without expecting anything in return.	0.94	True	0	-2.46
2	they feel accepted.	0.92	True	0	-1.76
3	they make up after a fight.	0.82	True	0	0.17
4	they are hugged.	0.88	True	0	-0.83
5	somebody confides in them.	0.76	True	0	0.44
6	they feel connected to God.	0.83	True	0	0.68
7	they play sports.	0.12	False	0	-0.22
8	the sun is shining.	0.23	False	0	1.02
9	someone tells them what is best for them.	0.25	False	0	1.26
10	they feel appreciated.	0.91	True	0	-1.68
11	they win on the lottery.	0.21	False	0	0.37
12	they feel part of a team.	0.67	True	0	1.56
13	someone understands them.	0.89	True	0	-1.32
14	someone is sexually attracted to them.	0.62	True	0	1.86
15	they get gifts.	0.79	True	0	-0.06
16	they spend time with their child(ren).	0.94	True	0	-1.80
17	someone helps them.	0.78	True	0	0.15
18	someone follows up to ask how a problem turned out.	0.67	True	0	1.16
19	they make love.	0.94	True	0	-1.49
20	someone cares for them.	0.97	True	0	-3.18
21	someone forgives them for something they did wrong.	0.83	True	0	-0.25
22	their pets are happy to see them.	0.97	True	0	-2.81
23	they attend a religious ceremony (e.g., church, temple, synagogue, mosque).	0.51	True	0.02	2.89
24	they attend sporting events of their favorite team.	0.20	False	0	0.49
25	they solve a difficult problem.	0.14	False	0	0.10
26	someone else wants to know where they are at all times.	0.44	False	0.03	2.65
27	they are made to feel special.	0.96	True	0	-2.64
28	someone can immediately tell what is on their mind.	0.62	True	0	1.63
29	someone does something nice for them unexpectedly.	0.92	True	0	-1.93
30	someone is supportive of their life goals.	0.92	True	0	-1.59
31	a child snuggles up to them.	0.93	True	0	-2.05
32	they are included in activities.	0.76	True	0	0.19
33	they go out for a walk.	0.15	False	0	0.12
34	they receive a compliment.	0.72	True	0	0.96
35	someone is insisting to spend all of their time with them.	0.68	True	0	1.42
36	they are told that they are loved.	0.95	True	0	-2.16
37	someone shows compassion towards them in difficult times.	0.94	True	0	-2.08
38	someone celebrates their accomplishments.	0.88	True	0	-1.04
39	they spend quality time with someone.	0.90	True	0	-1.49
40	they feel completely comfortable around someone.	0.89	True	0	-1.11
41	they hear or sing their country's national anthem.	0.30	False	0	1.80
42	they eat their favorite food.	0.27	False	0	1.15
43	someone is possessive about them.	0.37	False	0	1.60
44	someone is polite to them.	0.38	False	0.42	3.50
45	they can share their opinions without being judged.	0.81	True	0	0.23
46	they experience an act of kindness.	0.84	True	0	-0.50
47	they spend time with their friends.	0.89	True	0	-0.97
48	they are the recipient of physical affection.	0.90	True	0	-1.52
49	they feel close to nature.	0.46	False	0.49	3.36
50	a group recognizes their contribution.	0.74	True	0	0.62
51	they get a good night's sleep.	0.16	False	0	0.24
52	someone tries to change their behavior to be healthier.	0.34	False	0.02	2.33
53	they are recipients of gratitude.	0.79	True	0	0.07
