# Supplementary material for: Do People Agree on What Makes One Feel Loved? A Cognitive Psychometric Approach to the Consensus on Felt Love
Source: PLoS One. 2016 Apr 1;11(4):e0152803. doi: 10.1371/journal.pone.0152803 (PMC4818109; doi:10.1371/journal.pone.0152803)
Supplement: S2 Table — (PDF) [file pone.0152803.s002.pdf]

Do people agree on what makes one feel loved? A cognitive psychometric approach to the consensus on felt love

Zita Oravecz[1, *], Chelsea Muth[1], and Joachim Vandekerckhove[2]

1 Human Development and Family Studies, The Pennsylvania State University, State College, PA, USA
2 Cognitive Sciences, University of California, Irvine, Irvine, CA, USA

*** zita@psu.edu**

Supporting Information

S2 Table

Table. Model parameters regressed on a set of predictors.

S2 Table. Regression coefficients: all model parameters regressed on a set of predictors.
The first column shows the ECM parameter name, second column is the explanatory variable name, third column is the posterior mean estimate of the corresponding regression coefficient, fourth column is its posterior standard deviation and the last column shows the probability of the regression coefficient being smaller than 0. In this last column, values close to 1 indicate substantial probability that the likely values for this parameter are negative, while values close to 0 in the last column indicate small probabilities of the coefficient being negative, which in turn means large probabilities of the regression coefficient being positive.

**Parameter**	**Predictor**	**mean**	**std**	**p(<0)**
Consensus knowledge	Intercept	0.43	0.28	0.07
Consensus knowledge	Gender (1: male)	-0.16	0.15	0.86
Consensus knowledge	In a relationship	-0.03	0.15	0.58
Consensus knowledge	Age	0.20	0.14	0.09
Consensus knowledge	Household size	-0.27	0.15	0.96
Consensus knowledge	Nr. of siblings	0.12	0.15	0.20
Guessing "True"	Intercept	-0.15	0.29	0.69
Guessing "True"	Gender (1: male)	-0.46	0.22	0.98
Guessing "True"	In a relationship	0.41	0.22	0.03
Guessing "True"	Age	-0.22	0.21	0.87
Guessing "True"	Household size	-0.06	0.22	0.61
Guessing "True"	Nr. of siblings	0.22	0.21	0.14
Willingness to guess	Intercept	3.20	0.27	0
Willingness to guess	Gender (1: male)	0.35	0.25	0.08
Willingness to guess	In a relationship	-0.24	0.26	0.82
Willingness to guess	Age	0.02	0.26	0.47
Willingness to guess	Household size	0.50	0.28	0.03
Willingness to guess	Nr. of siblings	0.36	0.27	0.09
